# Identification and functional characterization of the sulfate transporter gene *GmSULTR1;2b* in soybean

**DOI:** 10.1186/s12864-016-2705-3

**Published:** 2016-05-20

**Authors:** Yiqiong Ding, Xiaoqiong Zhou, Li Zuo, Hui Wang, Deyue Yu

**Affiliations:** National Key Laboratory of Crop Genetics and Germplasm Enhancement, National Center for Soybean Improvement, Nanjing Agricultural University, Weigang No. 1, Nanjing, 210095 China

**Keywords:** Soybean, Sulfate transporter, *GmSULTR1;2b*, Low-sulfur stress, Sulfate uptake

## Abstract

**Background:**

Soybean is a major source of oil and protein in the human diet and in animal feed. However, as soybean is deficient in sulfur-containing amino acids, its nutritional value is limited. Increasing sulfate assimilation and utilization efficiency is a valuable approach to augment the concentration of sulfur-containing amino acids in soybean seeds, and sulfate transporters play important roles in both sulfate uptake and translocation within plants.

**Results:**

In this study, we isolated and characterized a soybean sulfate transporter gene: *GmSULTR1;2b*. The gene was found to be specifically expressed in root tissues and induced by low-sulfur stress. In addition, *GmSULTR1;2b* expression in yeast could complement deficiency in the sulfate transporter genes *SUL1* and *SUL2*. Under +S conditions, *GmSULTR1;2b*-overexpressing tobacco plants accumulated higher levels of organic matter and exhibited enhanced biomass and seed weight compared to control plants. Under -S conditions, acclimation of *GmSULTR1;2b*-overexpressing plants was much better than control plants. *GmSULTR1;2b*-overexpressing tobacco seedlings showed better tolerance to drought stress than the control plants, but no significant difference was observed under salt stress. Transcriptome analysis revealed 515 genes with at least a 2-fold change in expression in the leaves of tobacco plants overexpressing *GmSULTR1;2b*. Of these, 227 gene annotations were classified into 12 functional categories associated with 123 relevant pathways, including biosynthesis and metabolism-related proteins, stress-related proteins, and transporters.

**Conclusions:**

The findings reported here indicate that the increased biomass and seed yield observed in transgenic tobacco plants could have resulted from greater nutrient uptake and transport capability as well as enhanced development and accumulation of organic matter. Taken together, our results indicate that *GmSULTR1;2b* plays an important role in sulfur uptake and could alter the sulfur status of plants. Our study suggests that overexpressing *GmSULTR1;2b* may enhance plant yield under +S conditions, reduce plant production loss under -S conditions, and improve plant tolerance to sulfur deficiency stress.

**Electronic supplementary material:**

The online version of this article (doi:10.1186/s12864-016-2705-3) contains supplementary material, which is available to authorized users.

## Background

Soybean is a major source of oil and protein in the human diet and in animal feed. However, as soybean is deficient in sulfur-containing amino acids (methionine, cystine and cysteine), its nutritional value is limited. The N/S ratio of soybean seed is considered an indicator of protein quality. Marsolais et al. [1] reported that major seed storage proteins deficiency was associated with a nearly two-fold increase in sulfur-containing amino acid content in genetically related lines of common bean. Accordingly, increasing the content of sulfur-containing amino acids in seed proteins is a target of soybean breeding. Sulfur deficiency in soil is one reason for the decreased synthesis of sulfur-containing storage proteins [2], and the ability of soils to retain and release sulfur to crops is reduced with increases in planting density. This situation has led to a growing input of high-analysis low-sulfur-containing fertilizers and has globally resulted in widespread areas of sulfur deficiency [3]. In China, sulfur deficiency in soil has been reported in south China, northeast China, Shandong Province, and Shanxi Province [4]. For these reasons, the mining and characterization of genes that could genetically alter sulfate assimilation and utilization efficiency is an important approach for increasing the amount of sulfur-containing amino acids in soybean seeds.

Plants carry out inorganic sulfate assimilation, which involves complicated reduction and assimilatory reactions, by utilizing organic sulfur compounds, such as sulfur-containing amino acids, co-enzymes with iron-sulfur centers, thiamine, lipoic acid, coenzyme A, many secondary metabolites (e.g., glucosinolates). The first step in the assimilatory process is the uptake of sulfate from the environment by a plant proton/sulfate co-transporter in the plasma membrane of root cells. Various sulfate transporters with different affinities, capacities and cell type-specific localizations are encoded by a family of genes, and these transporters are responsible for both the absorption of sulfate and its accumulation within cells [5–7].

Sulfate transporters (SULTRs) are encoded by a large gene family, comprising 12 genes in *Arabidopsis*, 10 in wheat, 12 in rice and 16 in *Populus stremula* × *P. alba* [5, 8–10], that can be subdivided into four groups with divergent functions [10]. Group 1 SULTRs are high-affinity transporters, group 2 low-affinity transporters, and group 4 vacuolar sulfate exporters. Group 3 SULTRs are the most diverse, encoding transporters of plastid membranes and symbiosome membranes as well as others with specific or unknown functions [11]. All known sulfate transporters have 12 membrane-spanning domains (MSDs) and a sulfate transporter anti-sigma (STAS) domain at their carboxy-terminus [12]. The STAS domain of AtSULTR1;2 has been reported to facilitate localization of the transporter at the plasma membrane and to influence the kinetic properties of the catalytic domain [13]; the STAS domain is also involved in regulating the activity of O-acetyl-serine (thiol) lyase (OASTL) [14].

The SULTR family has been well characterized in *Arabidopsis*. The high-affinity transporters *AtSULTR1;1* and *AtSULTR1;2* co-localize in the root and are responsible for the uptake of sulfate from the soil [15]; *AtSULTR1;3* is localized in the phloem and is involved in the source-to-sink transport of sulfate [16]. Low-affinity sulfate transporters *AtSULTR2;1* and *AtSULTR2;2* mediate the translocation of internal sulfate within plants and are involved in regulating vascular sulfate transport [17, 18]; in addition, *AtSULTR2;1* is a target gene of miR395 [19, 20]. *AtSULTR3;5* is co-expressed with *AtSULTR2;1* and enhances root-to-shoot sulfate transport activity [21]. Lastly, *AtSULTR4;1* and *AtSULTR4;2* localize to the tonoplasts and mediate the efflux of sulfate from the vacuolar space into the cytoplasm [22].

Expression of sulfate transporters can be affected by the sulfur status of the plant. In poplar, responses to early sulfur deficiency include increases in the expression of *PtaSULTR1;1* and *miRNA395* [23], with long-term deficiency enhancing the expression of *PtaSULTR1;2* and *PtaSULTR4;2* [23]. Other responses involve *TaeSultr1;1*, *TaeSultr2;1*, and *TaeSultr4;1* expression in wheat [5] and *AtSULTR2;1* expression in xylem parenchyma and pericycle cells in *Arabidopsis* roots [18].

To date, there have been some studies about sulfate transporters in soybean rhizobia. GmN70 transcripts appeared in the root just before nodule emergence [24]; the GmN70 sequence (D13505) is part of that of GmSULTR2;3 (Glyma18g02240). Additionally, Clarke et al. [25] confirmed the involvement of soybean sulfate transporters in rhizobia symbiosis. Moreover, SST1 transports sulfate from the plant cell cytoplasm to intracellular rhizobia in the model legume *Lotus japonicus* [26].

Although SULTR genes have been characterized in other plants, there are few reporters about soybean sulfate transporters. In this work, we performed a comprehensive investigation of the soybean SULTR gene family using phylogenetic and expression analyses. Furthermore, we isolated soybean *GmSULTR1;2b* and characterized the function in soybean tissues, yeast cells and transgenic tobacco plants. We also attempted to explain the mechanism by which GmSULTR1;2b transports sulfate in transgenic tobacco plants using microarray analysis. This work presents an examination of the SULTR gene family at the genomic level, and the results will provide a basis for further investigation of the functions of soybean SULTR genes.

## Results

### Identification and phylogenetic and expression analyses of SULTR genes

**Fig. 1:**
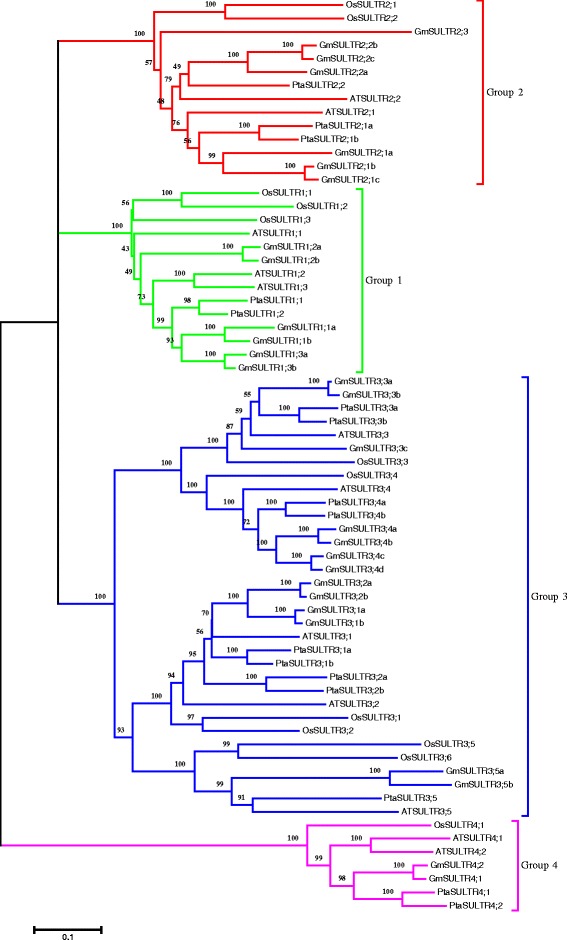
Phylogenetic analysis of predicted sulfate transporter amino acid sequences from soybean. A phylogenetic tree was generated using putative amino acid sequences with MEGA 5 and the neighbor-joining method. Bootstrap values support percentages from 1000 replicate trees. The scale bar indicates 0.1 substitutions per site in the amino acid sequence. The putative protein sequence accession numbers are provided in Additional file 1: Table S2

Twelve *Arabidopsis* SULTRs were used as query sequences for BLASTN searches of the soybean database in Phytozome (http://www.phytozome.net) with default parameters; redundant sequences were manually discarded. Twenty-eight putative SULTR genes, located on 15 chromosomes, were identified in the soybean genome (Additional file 1: Table S1). Their proteins all contain STAS domain in the C-terminal region, which is critical for sulfate transporter activity and stability. To gain insight into the biological functions of these genes, a phylogenetic tree was constructed based on a full-length amino acid alignment of the SULTRs (Fig. 1 and Additional file 1: Table S2), including 28 putative soybean SULTR sequences, 12 *Arabidopsis thaliana* SULTR sequences, 12 *Oryza sativa* SULTR sequences, and 16 *Populus tremula* × *P. alba* SULTR sequences. Following Takahashi et al. [10, 11], the 28 putative soybean SULTR proteins were classified into 4 groups based on phylogenetic analysis: 6 sequences in group 1, 7 in group 2, 13 in group 3, and 2 in group 4 (Fig. 1). Therefore, the putative soybean SULTR genes are likely to share similar functions with other members of the same subfamily. These soybean genes were named corresponding to homologous genes from other species.

**Fig. 2:**
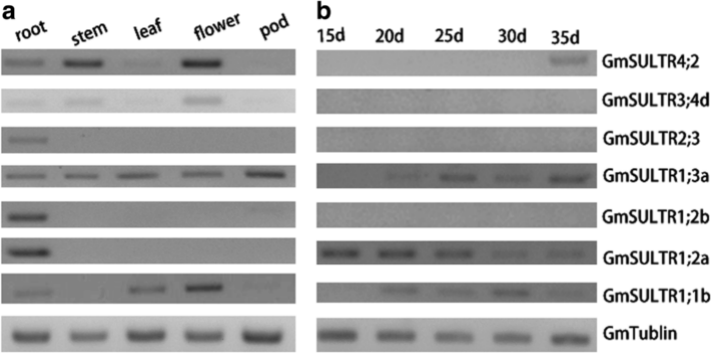
Expression profiling of 7 putative soybean SULTR genes. (**a**) Expression profiling of 7 putative soybean SULTR genes in different tissues using semi-quantitative RT-PCR. (**b**) Expression profiling of 7 putative soybean SULTR genes at different days after flowering using semi-quantitative RT-PCR

Based on *in silico* expression analysis, the 28 putative *SULTR* genes exhibit different tissue expression patterns (Additional file 1: Figure S1). Many of the soybean SULTR transcripts were abundant in the root, stem, and seedling. To validate the reliability of the expression profiles obtained from *in silico* expression analysis, we conducted RT-PCR for a subset of 7 genes from 4 groups (Fig. 2). As shown in Fig. 2 and Additional file 1: Figure S1, expression of most of the genes was the same across the two platforms. Among the soybean SULTR genes, *GmSULTR1;2b* (group 1) was primarily expressed in the root (Fig. 2 and Additional file 1: Figure S1); thus, we further cloned and analyzed this gene.

### *GmSULTR1;2b* is specifically expressed in roots and is induced by sulfur deficiency

The genomic sequence of *GmSULTR1;2b*, located on chromosome 15, is 4759 bp and contains 13 exons and 12 introns. The *GmSULTR1;2b* ORF is 1980 bp and encodes a 659-amino acid polypeptide, with a calculated protein molecular mass of 72.19 kDa and a predicted pI of 9.84. The GmSULTR1;2b protein contains SULTR conserved domains (the STAS domain) and 12 predicted MSDs in the N-terminal region (Additional file 1: Figure S2). A conserved motif (DLxAGLTxAxLxIPQxIAYAxLAxLxPxYGLYSSFxPxxIYxxMGT/SSR) [27] with a significant number of polar residues is located between MSD 1 and MSD 2. GmSULTR1;2b is 95 % identical to GmSULTR1;2a. GmSULTR1;2b is also highly homologous to other plant high-affinity sulfate transporter proteins (Additional file 1: Figure S2), which are specifically expressed in the root and induced by sulfur deficiency, sharing 89 % identity with StSULTR1 (*Stylosanthes hamata*, CAA57710), 90 % with StSULTR2 (*S. hamata*, CAA57711), 87 % with BjSULTR1;2b (*Brassica juncea*, AFX60924) and 83 % with AtSULTR1;2 (*A. thaliana*, AEE36056).

The expression positions of *GmSULTR1;2b* were localized using onion epidermal cells as a transient expression system. The coding region of *GmSULTR1;2b* fused with GFP was controlled by the CaMV 35S promoter. As shown in Additional file 1: Figure S3, the *GmSULTR1;2b*–GFP fusion protein was localized to the plasma membrane of onion epidermal cells, whereas the control (35S:GFP) was distributed throughout the entire cell.

**Fig. 3:**
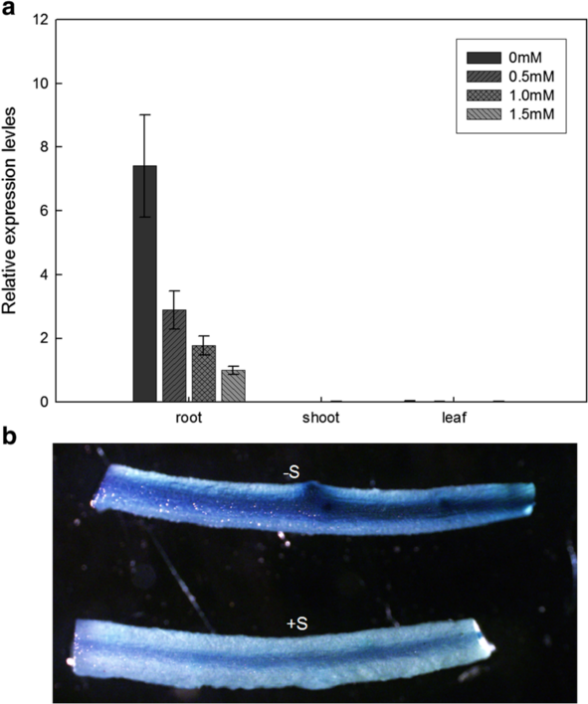
*GmSULTR1;2b* gene expression pattern in response to sulfur availability. (**a**) Real-time RT-PCR of the expression level of *GmSULTR1;2b* in different tissues of soybean plants treated for 10 days with 0 mM (−S) or 0.5, 1.0, and 1.5 mM MgSO_4_ (+S). (**b**) GUS staining of transgenic soybean hairy roots containing the *GmSULTR1;2b* promoter::GUS fusion protein, which was transformed into soybean cotyledons by A. rhizogenes. Error bars represent the standard deviation

The expression levels of *GmSULTR1;2b* in response to sulfur deprivation were analyzed by quantitative real time PCR (qRT-PCR) analysis. As shown in Fig 3a, expression was clearly up-regulated under sulfur deprivation conditions and increased with a decrease in the sulfate level in the soybean root. The expression level of *GmSULTR1;2b* under sulfate deficient conditions (0 mM MgSO_4_) was 7-fold higher than that under sulfate sufficient conditions (1.5 mM MgSO_4_; Fig. 3a). However, expression levels were unchanged in the soybean shoot and leaf. These results suggest that *GmSULTR1;2b* expression in the root is mediated by the sulfur status of the culture medium.

To further confirm root expression of *GmSULTR1;2b*, a 2259 bp fragment (data not shown) upstream of the *GmSULTR1;2b* translation start site (p*GmSULTR1;2b*) was amplified and fused to *GUS*. The construct was transformed into soybean hairy roots via *Agrobacterium rhizogenes*-mediated transformation, and hairy roots carrying p*GmSULTR1;2b*::GUS were stained for GUS activity. The GUS activity of p*GmSULTR1;2b*::GUS was higher under -S conditions compared to +S conditions (Fig. 3b), consistent with the expression patterns revealed by qRT-PCR (Fig. 3a). Therefore, the *GmSULTR1;2b* promoter can drive *GUS* expression in the root and is induced by low-sulfur stress.

### The *GmSULTR1;2b* protein exhibits sulfate transport activity

**Fig. 4:**
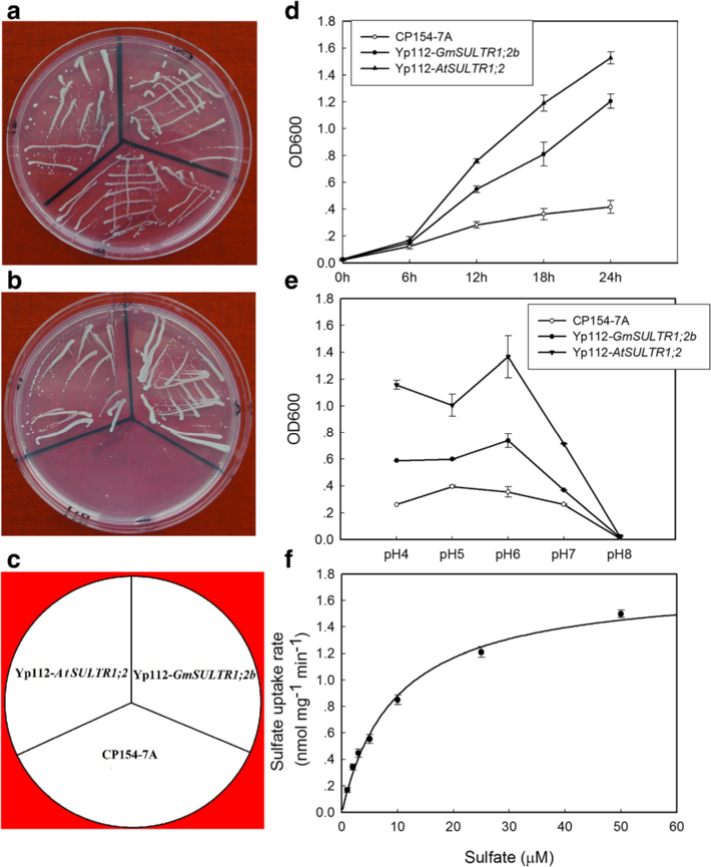
Functional expression of *GmSULTR1;2b* in yeast. (**a**) YNB solid culture medium containing 0.1 mM homocysteine. (**b**) YNB solid culture medium containing 0.1 mM sodium sulfate. (**c**) The position of each yeast strain on the plates. (**d**) Growth curves generated from a 24 h culture grown in YNB liquid culture medium containing 0.1 mM sodium sulfate. (**e**) Effects of culture medium pH on the growth of the three yeast strains grown in YNB liquid culture medium containing 0.1 mM sodium sulfate. (**f**) Uptake of ^35^S-labeled sulfate by Yp112-*GmSULTR1;2b* yeast cells. Sulfate uptake rates were calculated after transferring the cells to media containing different external sulfate concentrations at pH 6.0. Error bars represent the standard deviation. CP154-7A, CP154-7A mutant strain transformed with the empty vector (as a negative control); Yp112-*AtSULTR1;2*, CP154-7A transformed with Yp112-*AtSULTR1;2* (as a positive control); Yp112-*GmSULTR1;2b*, CP154-7A transformed with Yp112-*GmSULTR1;2b*

To evaluate the sulfate transport activity of *GmSULTR1;2b*, we performed a complementation analysis using the CP154-7A yeast mutant strain, which is unable to grow on medium containing low concentrations of sulfate as the sole sulfur source because it carries two defective sulfate transporter genes (*SUL1* and *SUL2*). This mutant yeast strain was transformed with *GmSULTR1;2b*, *AtSULTR1;2* (as a positive control), or the p112A1NE empty expression vector (as a negative control) and grown for 3 days on yeast nitrogen base (YNB) medium containing 0.1 mM sodium sulfate or 0.1 mM homocysteine as the sole sulfur source. Mutant cells expressing Yp112-*GmSULTR1;2b*, Yp112-*AtSULTR1;2* or the empty vector all grew well with 0.1 mM homocysteine, but only the cells expressing Yp112-*GmSULTR1;2b* or Yp112-*AtSULTR1;2* grew well on medium containing 0.1 mM sodium sulfate as the sole sulfur source (Fig. 4a, b and c). These results suggest that the protein encoded by *GmSULTR1;2b* is able to complement yeast cells defective in sulfate transport.

Although cells harboring the three constructs could grow in YNB medium containing 0.1 mM sodium sulfate, the mutant cells expressing Yp112-*GmSULTR1;2b* or Yp112-*AtSULTR1;2* grew much better than those expressing the empty vector (Fig. 4d), and the mutants expressing Yp112-*GmSULTR1;2b* or Yp112-*AtSULTR1;2* grew much better at pH 4 to 6 than at pH 7 to 8 (Fig. 4e). Indeed, the growth rates of both strains decreased markedly when the pH was increased from 6 to 8. Thus, the optimum pH for the mutant cells with Yp112-*GmSULTR1;2b* or Yp112-*AtSULTR1;2* is slightly acidic, at pH 6. Cells carrying Yp112-*AtSULTR1;2* grew notably better than those carrying Yp112-*GmSULTR1;2b* at pH 4 to 6, though the growth rate of the two strains was similar at pH values of 7 and 8. These results indicate that the sulfate transporters have different proton (H^+^) dependence or that a change in affinity occurs at different external pH values. To determine the kinetic properties of the GmSULTR1;2b transporter, sulfate uptake rates in complemented CP154-7A yeast cells were measured and found to be between 30 and 90 s after suspending the cells in ^35^S labeled medium. These data indicate that sulfate uptake mediated by the GmSULTR1;2b transporter follows Michaelis-Menten kinetics, with a *K*_*m*_ of 9.5 ± 1.7 μM (Fig. 4f).

### *GmSULTR1;2b* confers tolerance to transgenic tobacco plants under sulfur deficiency stress

**Fig. 5:**
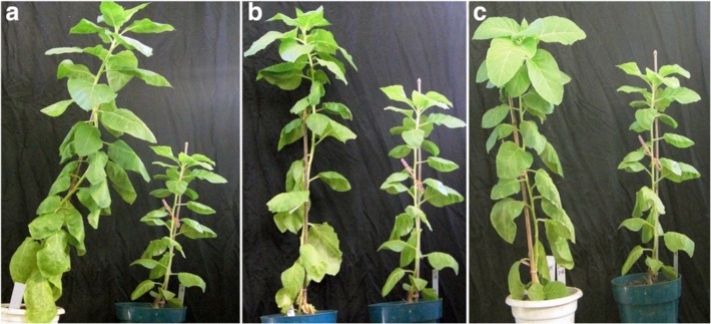
Phenotype of *GmSULTR1;2b*-overexpressing tobacco plants. (**a**) The *GmSULTR1;2b*-overexpressing S22 line plant grown under normal conditions in soil is shown on the left and the control plant (CK) on the right. (**b**) The S26 line plant is shown on the left and the CK plant on the right. (**c**) The S29 line plant is shown on the left and the CK plant on the right

**Fig. 6:**
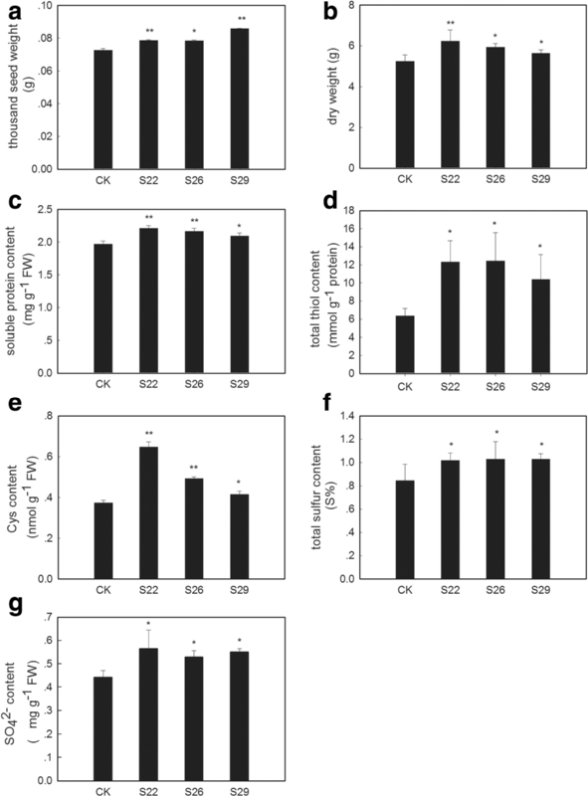
Biomass, yield and content of sulfur-containing compounds in *GmSULTR1;2b*-overexpressing tobacco plants grown under +S conditions. (**a**) Thousand seed weight of seeds from *GmSULTR1;2b*-overexpressing tobacco plants and control plants grown in soil. (**b**) Dry weights of 2-month-old whole *GmSULTR1;2b*-overexpressing tobacco plants and whole control plants grown under +S (1.5 mM MgSO_4_) conditions. (**c**) Soluble protein content in the functional leaves of 2-month-old *GmSULTR1;2b*-overexpressing tobacco plants and control plants grown under +S conditions. (**d**) Total thiol content in the functional leaves of 2-month-old *GmSULTR1;2b*-overexpressing tobacco plants and control plants grown under +S conditions. (**e**) Cys content in the functional leaves of *GmSULTR1;2b*-overexpressing tobacco plants and control plants grown under +S conditions. (**f**) Total sulfur content in the functional leaves of 2-month-old *GmSULTR1;2b*-overexpressing tobacco plants and control plants grown under +S conditions. (**g**) Sulfate ion content in whole roots of 2-month-old *GmSULTR1;2b*-overexpressing tobacco plants and control plants grown under +S conditions. Three biological replicates were performed. Error bars represent the standard deviation. Asterisks (*) indicate significant differences at *P* < 0.05 (Student’s *t*-test), and double asterisks (**) indicate significant differences at *P* < 0.01 (Student’s *t*-test). CK, tobacco plant transformed with the pMDC83 empty vector as a control; S22, S26 and S29, tobacco plants transformed with *GmSULTR1;2b*, FW fresh weight

To gain further insight into the function of *GmSULTR1;2b*, a construct containing the *GmSULTR1;2b* ORF driven by 35S promoter was transferred into tobacco plants; after PCR analysis (Additional file 1: Figure S4) and Southern blotting of all transformed plants, three independent lines, namely, S22, S26 and S29, with one copy of the *GmSULTR1;2b* gene, were selected for further study (data not shown). We found that the T_2_*GmSULTR1;2b*-overexpressing tobacco plants grew better than the control plants (Fig. 5). Moreover, the seed weights of lines of S22, S26 and S29 were significantly improved, with the average increasing by 11 % (Fig. 6a). These findings indicate that *GmSULTR1;2b* overexpression could enhance seed yield.

To obtain additional details, 2 month-old tobacco plants of lines S22, S26 and S29 were examined with regard to biomass and the content of sulfur-containing compounds. Under +S conditions, the tobacco plants overexpressing *GmSULTR1;2b* accumulated more biomass compared to the control plants (Fig. 6b), with an average increase of 13 %, showing that the former maintained better growth and development. Additionally, the soluble protein content (Fig. 6c) and total thiol content (Fig. 6d) of leaves were enhanced in the *GmSULTR1;2b*-overexpressing plants compared to the control plants, with average increases of 9 and 83 %. Among total thiols, the Cys content (Fig. 6e) of leaves was enhanced. These findings indicate that overexpression of GmSULTR1;2b could enhance the content of organic matter, especially organic sulfur including sulfur-containing amino acids. In addition, the total sulfur content of whole plants (Fig. 6f) was increased in the *GmSULTR1;2b*-overexpressing plants compared to the control plants. In contrast, the total carbon (C) and nitrogen (N) contents of the *GmSULTR1;2b*-overexpressing plants were not significantly changed compared to the control plants (Table 1). The SO_4_^2−^ content of roots was also increased significantly, by an average of 23 % (Fig. 6g). These results indicate that overexpression of *GmSULTR1;2b* enhanced the sulfur absorption capacity of the plants.

**Table 1:** Contents of C, N, and S in *GmSULTR1;2b*-overexpressing tobacco plants and control plants

	Lines	%C	%N	%S
Shoot	CK	39.9 ± 0.11	1.47 ± 0.11	0.375 ± 0.05
	S22	39.6 ± 0.36	1.44 ± 0.04	0.461 ± 0.03^*^
	S26	40.0 ± 0.06	1.48 ± 0.05	0.481 ± 0.06^*^
	S29	39.6 ± 0.21	1.48 ± 0.04	0.441 ± 0.01^*^
Root	CK	40.9 ± 1.08	1.81 ± 0.16	0.471 ± 0.09
	S22	41.9 ± 0.83	1.77 ± 0.08	0.555 ± 0.03^*^
	S26	41.8 ± 0.20	1.73 ± 0.05	0.548 ± 0.09^*^
	S29	41.5 ± 0.26	1.81 ± 0.06	0.59 ± 0.02^*^

CK, tobacco plant transformed with the pMDC83 empty vector as a control; S22 S26 and S29, tobacco plants transformed with *GmSULTR1;2b*. *: significant at 0.05 (Student’s *t*-test, *n* = 3)

**Fig. 7:**
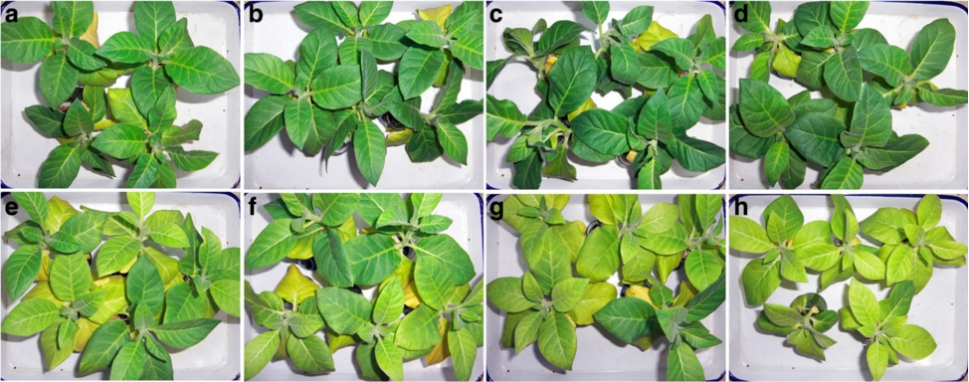
Phenotype of *GmSULTR1;2b*-overexpressing tobacco plants under +S and -S conditions. (**a**) Plants of the line S22 grown under +S conditions (1.5 mM MgSO_4_) for 2 weeks. (**b**) Plants of the line S26 grown under +S conditions for 2 weeks. (**c**) Plants of the line S29 grown under +S conditions for 2 weeks. (**d**) Control plants grown under +S conditions for 2 weeks. (**e**) Plants of the line S22 grown under -S conditions (0 mM MgSO_4_) for 2 weeks. (**f**) Plants of the line S26 grown under -S conditions for 2 weeks. (**g**) Plants of the line S29 grown under -S conditions for 2 weeks. (**h**) Control plants grown under -S conditions for 2 weeks

**Fig. 8:**
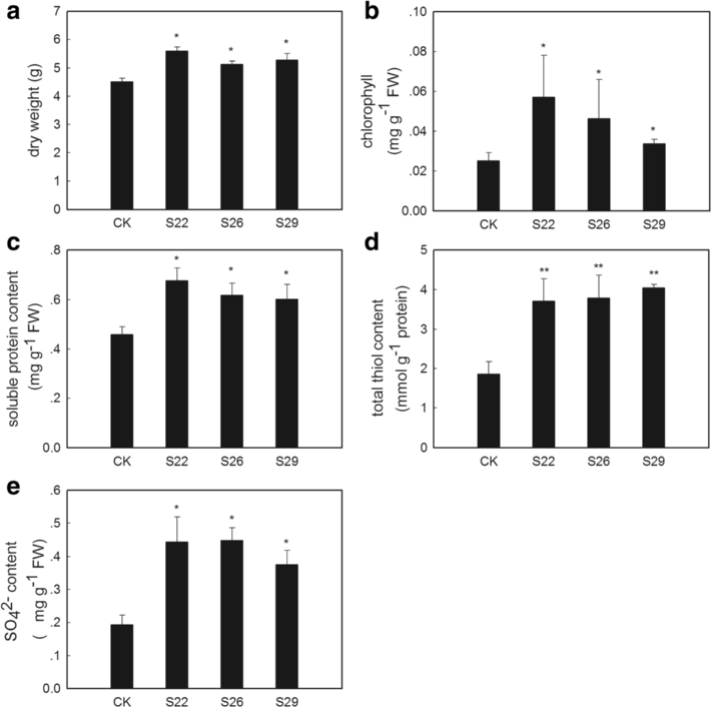
Biomass and content of sulfur-containing compounds in *GmSULTR1;2b*-overexpressing tobacco plants grown under -S conditions. (**a**) Dry weights of 2-month-old whole *GmSULTR1;2b*-overexpressing tobacco plants and whole control plants under -S (0 mM MgSO_4_) conditions. (**b**) Chlorophyll content of the leaves of *GmSULTR1;2b*-overexpressing tobacco plants and control plants grown under -S conditions for 2 weeks. (**c**) Soluble protein content in the functional leaves of 2-month-old *GmSULTR1;2b*-overexpressing tobacco plants and control plants grown under -S conditions. (**d**) Total thiol content in the functional leaves of 2-month-old *GmSULTR1;2b*-overexpressing tobacco plants and control plants grown under -S conditions. (**e**) Sulfate ion content in whole roots of 2-month-old *GmSULTR1;2b*-overexpressing tobacco plants and control plants grown under -S conditions. Three biological replicates were performed. Error bars represent the standard deviation. Asterisks (*) indicate significant differences at *P* < 0.05 (Student’s *t*-test), and double asterisks (**) indicate significant differences at *P* < 0.01 (Student’s *t*-test). CK, tobacco plant transformed with the pMDC83 empty vector as a control; S22, S26 and S29, tobacco plants transformed with *GmSULTR1;2b*, FW fresh weight

Under -S conditions, the *GmSULTR1;2b*-overexpressing plants showed chlorosis but not as severe as the controls, whereas the control plants displayed severe chlorosis symptoms after 2 weeks of growth (Fig. 7). Under sulfur deficiency stress, the tobacco plants overexpressing *GmSULTR1;2b* accumulated more biomass compared to the control plants, on average more than 18 % (Fig. 8a). The chlorophyll content of the *GmSULTR1;2b*-overexpressing tobacco plants was significantly higher than that of the control tobacco plants (Fig. 8b), though there were no significantly differences between the *GmSULTR1;2b*-overexpressing and control plants under +S conditions (Additional file 1: Figure S5 and Fig. 7). Moreover, under the -S condition, the soluble protein and total thiol contents of the leaves were also an average of 37 and 107 % greater in the *GmSULTR1;2b*-overexpressing plants compared to the control plants (Fig. 8c and d), as was the SO_4_^2−^ content of the roots (an average of more than 119 %) (Fig. 8e). The SO_4_^2−^ content of the roots in the *GmSULTR1;2b*-overexpressing tobacco plants was not significantly changed under the -S condition compared to under the +S condition, whereas a substantial reduction in the control plants was observed (Figs. 6g and 8e). In addition, the differences under the -S condition were more obvious than under the +S condition, with the biomass and soluble protein and total thiol contents of the *GmSULTR1;2b*-overexpressing plants decreasing less than those of the control plants under the –S condition compared to that under the +S condition. These findings indicated that under -S conditions, acclimation by the *GmSULTR1;2b*-overexpressing plants was much better than that of the control plants. All these results indicate that overexpression of *GmSULTR1;2b* enhanced plant yield under +S conditions, reduced plant production loss under -S conditions, and improved plant tolerance to sulfur deficiency stress.

**Fig. 9:**
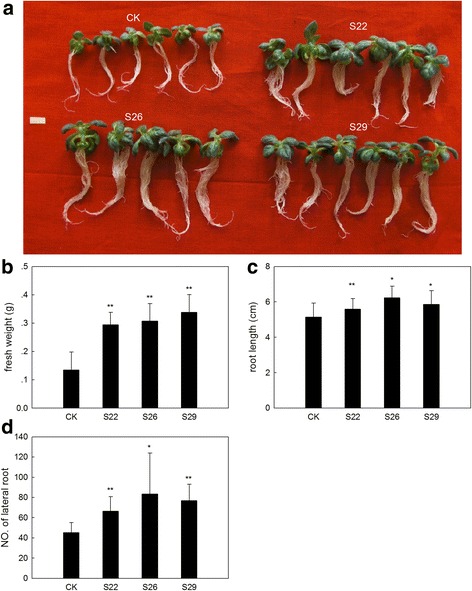
*GmSULTR1;2b*-overexpressing tobacco plants grown under 200 mM mannitol treatment. (**a**) Phenotypes of *GmSULTR1;2b*-overexpressing tobacco seedlings grown under 200 mM mannitol treatment for 4 weeks. (**b**) Fresh weight of *GmSULTR1;2b*-overexpressing tobacco seedlings grown under 200 mM mannitol treatment. (**c**) Root length of *GmSULTR1;2b*-overexpressing tobacco seedlings grown under 200 mM mannitol treatment. (**d**) Number of lateral roots of *GmSULTR1;2b*-overexpressing tobacco seedlings grown under 200 mM mannitol treatment. Ten biological replicates were performed. Error bars represent the standard deviation. Asterisks (*) indicate significant differences at *P* < 0.05 (Student’s *t*-test), and double asterisks (**) indicate significant differences at *P* < 0.01 (Student’s *t*-test). CK, tobacco plant transformed with the pMDC83 empty vector as a control; S22, S26 and S29, tobacco plants transformed with GmSULTR1;2b. Scale bar = 1 cm

**Fig. 10:**
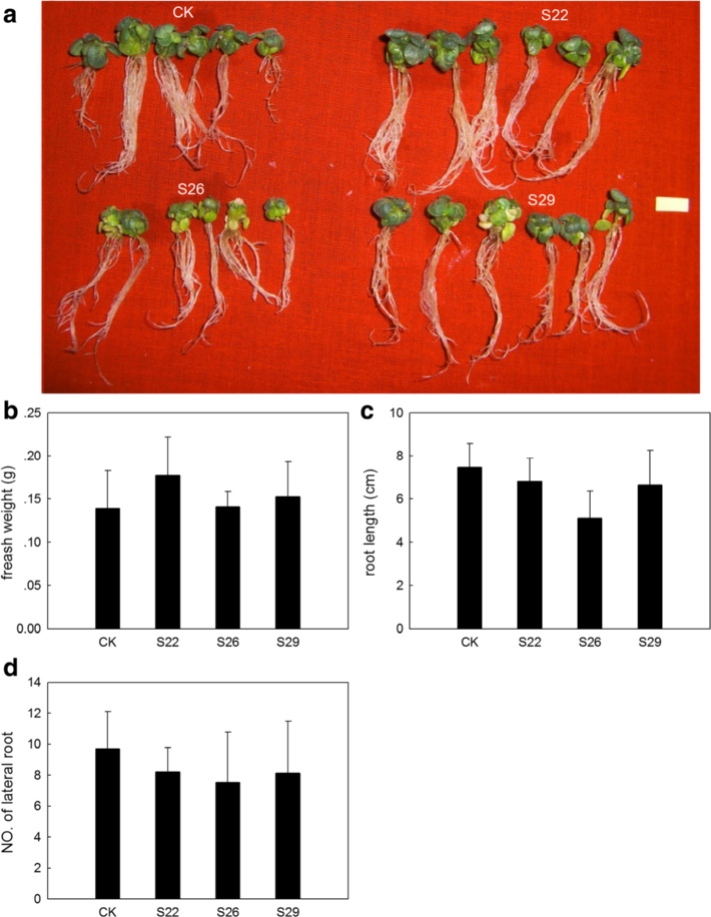
*GmSULTR1;2b*-overexpressing tobacco plants grown under 200 mM NaCl treatment. (**a**) Phenotypes of *GmSULTR1;2b*-overexpressing tobacco seedlings grown under 200 mM NaCl treatment for 4 weeks. (**b**) Fresh weight of *GmSULTR1;2b*-overexpressing tobacco seedlings grown under 200 mM NaCl treatment. (**c**) Root length of *GmSULTR1;2b*-overexpressing tobacco seedlings grown under 200 mM NaCl treatment. (**d**) Number of lateral roots of *GmSULTR1;2b*-overexpressing tobacco seedlings grown under 200 mM NaCl treatment. Ten biological replicates were performed. Error bars represent the standard deviation. Asterisks (*) indicate significant differences at *P* < 0.05 (Student’s *t*-test), and double asterisks (**) indicate significant differences at *P* < 0.01 (Student’s *t*-test). CK, tobacco plant transformed with the pMDC83 empty vector as a control; S22, S26 and S29, tobacco plants transformed with GmSULTR1;2b. Scale bar = 1 cm

Many studies have shown that sulfur-containing compounds are associated with stress. To address whether an increased total thiol content could enhance stress tolerance, we also subjected *GmSULTR1;2b*-overexpressing plants and control plants to drought stress (200 mM mannitol) and salt stress (200 mM NaCl) (Fig. 9a). Under drought stress, it showed that the fresh weight of *GmSULTR1;2b*-overexpressing tobacco seedlings were average 132 % more than that of control seedlings (Fig. 9b), the root length of *GmSULTR1;2b*-overexpressing tobacco seedlings were average 15 % longer than that of control seedlings (Fig. 9c), and the number of lateral root of *GmSULTR1;2b*-overexpressing tobacco seedlings were average 67 % more than that of control seedlings (Fig. 9d). However, under salt stress, there were no significant difference in fresh weight, root length and the number of lateral root between *GmSULTR1;2b*-overexpressing tobacco seedlings and control seedlings (Fig. 10). These results indicated that the *GmSULTR1;2b*-overexpressing tobacco seedlings showed better tolerance to drought stress than the control plants, though no significant difference under salt stress was observed.

### *GmSULTR1;2b* overexpression results in changes in the transcripts of genes related to metabolism and transporters in tobacco plants

**Fig. 11:**
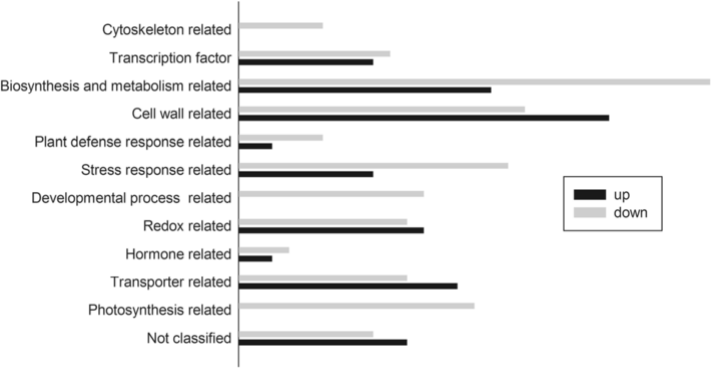
Overview of the microarray results for tobacco leaves showing ≥ 2.0-fold differences in expression. The number of genes in one of the 12 identified functional categories: cytoskeleton-related proteins; transcription factors; biosynthesis and metabolism-related proteins; cell wall-related proteins; plant defense response-related protein; stress-related proteins; development process-related proteins; redox-related proteins; hormone-related proteins; transporters; photosynthesis-related proteins; and unclassified functional proteins on the basis of gene ontology (http://bioinfo.capitalbio.com/mas3/) and gene annotations (NCBI and TIGR)

We compared mRNA levels in the leaves of control and *GmSULTR1.2b*-overexpressing tobacco plants grown in soil. A total of 515 probe sets exhibited at least a 2-fold change in expression in the leaves of the *GmSULTR1;2b*-overexpressing plants, with 205 being up-regulated and 310 down-regulated. Among these differentially expressed genes, 227 probe sets annotations were found in NCBI and TIGR (Additional file 2: Table S3), and 7 probe sets were assessed by qRT-PCR, confirming the reliability of the microarray results (Additional file 3: Tables S4 and S5). On the basis of gene annotations and gene ontology, these 227 probe sets were classified into 12 functional categories, including biosynthesis and metabolism-related proteins, cell wall-related proteins, stress-related proteins, transporters, redox-related proteins, transcription factors, photosynthesis-related proteins, and developmental process-related proteins (Fig. 11). Among them, the largest group of regulated transcripts in the *GmSULTR1;2b*-overexpressing plants was categorized as biosynthesis-and metabolism-related proteins. This group of transcripts included 43 related transcripts, among which the level of S-adenosylhomocysteine hydrolase (SAHH) was reduced. Among 23 genes related to transporters, the transcript levels of the NRAMP3 metal transporter, OPT3 oligopeptide transporter, mitochondrial phosphate transporter, potassium transporter, nitrite transporter, sugar transporter, and SULTR3;5 were up-regulated (Additional file 2: Table S3).

Furthermore, 219 differentially expressed probe sets were classified as associated with 123 relevant pathways described in Kyoto Encyclopedia of Genes and Genomes (KEGG). Information regarding these 123 pathways is shown in Additional file 4: Table S6. Thirteen significant functional hierarchical KEGG pathways (*p*-value <0.05) are shown in Table 2. Ten of the 13 KEGG pathways were found to be related to metabolic processes in *GmSULTR1;2b*-overexpressing plants compared to control plants. Among the 10 pathways, 12 probe sets are involved in photosynthesis, 9 in photosynthesis-antenna proteins, 10 in ascorbate and aldarate metabolism, 11 in cysteine and methionine metabolism, 3 in riboflavin metabolism, 13 in starch and sucrose metabolism, 9 in glutathione metabolism, 6 in inositol phosphate metabolism, 4 in aminobenzoate degradation and 17 in pentose and glucuronate interconversions (Table 2).

**Table 2:** The pathways and terms of related biological processes

Pathway ID	KEGG pathway terms	*p*-value	n/N
ko04978	Mineral absorption	0.001469	3/19
ko00195	Photosynthesis	1.05E-04	12/267
ko00196	Photosynthesis - antenna proteins	1.62E-04	9/162
ko04626	Plant-pathogen interaction	0.040562	19/219
ko04512	ECM-receptor interaction	0.002119	2/6
ko00053	Ascorbate and aldarate metabolism	1.67E-04	10/200
ko00270	Cysteine and methionine metabolism	0.009729	11/400
ko00740	Riboflavin metabolism	0.010042	3/37
ko00500	Starch and sucrose metabolism	0.012889	13/531
ko00480	Glutathione metabolism	0.01538	9/317
ko00562	Inositol phosphate metabolism	0.029619	6/192
ko00627	Aminobenzoate degradation	0.032258	4/99
ko00040	Pentose and glucuronate interconversions	2.29E-08	17/263

The functional hierarchy analysis was performed by KEGG (http://www.genome.jp/kegg) at a 0.05 level of significance. n/N represents the number of genes in the present study/the number of genes in the KEGG database

Altogether, overexpression of *GmSULTR1;2b* result in the genes categorized as biosynthesis- and metabolism-related proteins, transporters, photosynthesis-related proteins, and developmental process-related proteins were regulated. These genes may be the reason that overexpressing *GmSULTR1;2b* could enhance tobacco plant yield under +S conditions, reduce tobacco plant production loss under -S conditions.

## Discussion

Soybean is an ancient tetraploid, and the number of predicted coding genes in soybean is nearly twice that in *Arabidopsis* [28]. Due to genome duplications that occurred approximately 59 and 13 million years ago, nearly 75 % of soybean genes are present in multiple copies [29, 30]. Therefore, single-copy genes in *Arabidopsis* are generally represented by multiple copies in soybean. Accordingly, the number of sulfate transporter genes in soybean is more than the number of such genes in *Arabidopsis*. Indeed, there are 28 sulfate transporter genes in soybean but only 12 in *Arabidopsis*, 12 in *Oryza* and 16 in *Populus tremula × P. alba*. Most of the sulfate transporter genes in soybean exist as 2, 3 or 4 copies (Fig. 1), among which *GmSULTR1;2a* and *GmSULTR1;2b* are present as 2 copies. However, further research is required to determine the relative contribution of the 28 soybean sulfate transporter genes to overall sulfate transport and whether all copies are involved in sulfate acquisition, translocation and remobilization. In this study, we focused on *GmSULTR1;2b*.

### GmSULTR1;2b is a functional sulfate transporter and can be regulated by the sulfur status

Sulfate uptake into cells and efflux from cellular vacuoles is facilitated by multiple sulfate transporters, which in plants are H^+^/sulfate cotransporters depending on the proton motive force [31, 32]. The mutant yeast strain CP154-7A is defective for *SUL1* and *SUL2* genes and cannot transport sulfate under conditions of low sulfate availability [33]. Thus, the function of exogenous sulfate transporter genes can be demonstrated through complementation of this defect, and transport activity can be monitored using this yeast expression system [15–17, 34]. In the present study, CP154-7A harboring the *GmSULTR1;2b* cDNA was able to grow on YNB medium containing low concentrations of sulfate as the sole sulfur source, suggesting that *GmSULTR1;2b* encodes a functional sulfate transporter that can transport sulfate across the plasma membrane. The apparent uptake affinity of the high-affinity sulfate uptake system in plants operating at low sulfate concentrations has a *K*_m_ value that ranges from 1.5 to 11.5 μM, whereas the low-affinity system operating at high sulfate concentrations has a *K*_m_ value ranging from 400 to 1200 μM [15, 17, 21, 32]. Our kinetic analysis of the GmSULTR1;2b transporter revealed a *K*_m_ of 9.5 μM, with a high affinity for sulfate (Fig. 4f). Furthermore, the mutant strain expressing *GmSULTR1;2b* grew better at pH 4 to 6 than at pH 7 or 8. Altogether, we suggest that GmSULTR1;2b is a H^+^-dependent high-affinity transporter.

In plants, members of the sulfate transporter gene family are specifically expressed in different tissues and in response to reduced sulfur availability as well as other environmental conditions, and the protein products carry out different functions [5, 7, 35, 36]. In *Arabidopsis*, AtSULTR1;2 is localized to the root epidermis and cortex and is suggested to be the key root sulfate transporter facilitating the uptake of sulfate from the soil [34]. AtSULTR1;2 expression is induced by S deficiency, mainly regulated by metabolic demand, and controlled by photoperiod [37]. In soybean, the 28 putative soybean sulfate transporter genes show tissue-specific expression patterns. Of these genes, *GmSULTR1;2b* shares 83 % identity with AtSULTR1;2 and is specifically expressed in roots. Our results also demonstrate that expression of *GmSULTR1;2b* is induced by S deficiency, with expression that fluctuates in an alternating manner (data not shown). The growth curve of CP154-7A yeast cells expressing the *GmSULTR1;2b* cDNA was similar to that of CP154-7A expressing *AtSULTR1;2* cDNA (Fig. 4d and e). Taken together, we suggest that soybean GmSULTR1;2b might have functions similar to those of *Arabidopsis* AtSULTR1;2, i.e., in mediating the uptake of sulfate from the soil into plant roots.

### *GmSULTR1;2b* alters the sulfur status of overexpressing tobacco plants

Soil sulfur deficiency is a widespread limiting factor in crop production [38], and sulfur addition to the soil or foliar application is required to maintain or increase yield. Overall, an appropriate sulfur supply may help to maintain efficient photosynthesis and metabolism, enhance defense reactions and protein quality, and increase biomass production during periods of sulfur deficiency [39–41]. Genetic manipulation of sulfate transporters can improve sulfur utilization efficiency [35], and one advantage of improving the sulfate transporter system of plants would be to reduce the need to add sulfur fertilizer because these plants would be able to take up sulfur more efficiently and/or would possess the ability to reduce the loss of available sulfur from the soil. To date, soybean has remained one of the difficult crops to transform; indeed, compared to other crops, such as rice and corn, the transformation frequency of soybean is rather low [42]. Thus, it is very difficult for public research groups to transform genes into soybean, and researchers typically transform soybean genes into a model plant, such as tobacco or *Arabidopsis* for heterologous expression. Accordingly, we analyzed the function of *GmSULTR1;2b* through heterologous expression in tobacco plants and found that *GmSULTR1;2b-*overexpressing tobacco plants exhibited better growth and higher accumulation of biomass than the control plants. In addition, chlorosis in the control plants was more serious than in the plants overexpressing *GmSULTR1;2b* under conditions of sulfur deficiency, and the chlorophyll content increased compared to control plants under -S conditions. These results indicate that the *GmSULTR1;2b*-overexpressing plants were able to take up sulfur more efficiently. Thus, *GmSULTR1;2b* overexpression can improve the sulfur utilization efficiency and alter the sulfur status of transgenic plants.

C, N and S assimilation pathways constitute the most important pathways of plant primary metabolism, interacting in the synthesis of Cys and other important reduced sulfur-containing compounds, such as Met and GSH [43, 44]. C and N assimilation provides carbon skeletons and reduced nitrogen for the synthesis of amino acids and proteins, and S and N assimilation pathways are well coordinated and influence each other. Indeed, sulfur deficiency decreases nitrate uptake and the activity of nitrate reductase [45], and correspondingly, nitrogen deficiency reduces the activities of sulfate-assimilation enzymes and the transcript levels of relevant sulfate-assimilation genes [46]. In our study, *GmSULTR1;2b*-overexpressing tobacco plants showed enhanced accumulation of protein and sulfur-containing amino acids in leaves and an increase S content in aboveground parts. However, the total N and C contents were the same as those of control plants, suggesting that the concentrations of protein C and N increased while nonprotein C and N decreased.

### Possible mechanism by which *GmSULTR1;2b* alters the sulfur status in overexpressing tobacco plants

Due to the limitations of gene chip analysis, not every gene changes have been detected. Moreover, the tobacco genome sequencing results have not been published. Our gene annotation information was not complete. But we still have some useful findings. In our study, many genes related to transporters, such as *NRAMP* and *OPT*, were found to be regulated in tobacco plants overexpressing *GmSULTR1;2b. AtNRAMP3* and *AtNRAMP4* influence metal accumulation (transporting manganese, iron, and zinc), are involved different processes, including resistance to bacterial pathogens, support early development, and are required for optimal photosynthesis and growth [47–49]. *OPT3* can rescue copper-deficient and manganese-deficient yeast mutants and plays a critical role in the maintenance of whole-plant iron homeostasis and iron nutrition in developing *Arabidopsis* seeds [50, 51]. These findings suggest that *GmSULTR1;2b* may regulate other types of transporters to accumulate more nutrition, resulting in enhanced biomass and seed weight in tobacco plants overexpressing *GmSULTR1;2b*.

SAHH catalyzes the reversible hydrolysis of S-adenosyl-L-homocysteine to L-homocysteine and adenosine and is important for cell growth and regulating gene expression [52, 53]. Complete inactivation of SAHH activity in plants is embryonic lethal [54], and partial inactivation results in developmental malformations [55]. However, there are potential beneficial outcomes of the controlled down-regulation of *SAHH*, including the suppression of viral replication and enhancement of drought stress tolerance [55, 56]. In addition, down-regulation of *SAHH1* expression in yeast leads to both SAH accumulation and impacts on cellular lipid homeostasis [57]. In our study, the level of *SAHH* transcript was reduced in tobacco plants overexpressing *GmSULTR1;2b* compared to control tobacco plants. This result suggests that down-regulation of the *SAHH* gene may be a reason why the *GmSULTR1;2b*-overexpressing plants accumulated more sulfur-containing compounds (i.e., cysteine, thiol) than the control plants. In addition, the plants overexpressing *GmSULTR1;2b* exhibited enhanced drought stress tolerance. It has been reported that the application of sulfur fertilizer improves drought tolerance in plants [58], and it is possible that plants may down-regulate SAHH in response to *GmSULTR1;2b* overexpression not only to accumulate more sulfur-containing compounds but also to enhance growth and tolerance to biotic and abiotic stresses.

## Conclusion

In this study, we confirmed GmSULTR1;2b as a functional sulfate transporter that plays an important role in sulfate uptake and could alter the sulfur status of plants. We found that overexpression *GmSULTR1;2b* in tobacco plants could enhance yield under +S conditions, reduce production loss under -S conditions, and improve tolerance to sulfur deficiency stress. The mechanism by which increased biomass and seed yield in GmSULTR1;2b-overexpressing transgenic tobacco plants occurs could be due to greater nutrient uptake and transport capabilities as well as enhanced development and accumulation of organic matter, as indicated by microarray analysis.

## Methods

### Plant growth conditions

Soybean (*Glycine max* (L.) Merr. cv. N2899) seeds were germinated in vermiculite. When the cotyledons were fully expanded, the seedlings were transferred to MGRL [59] medium (1.75 mM sodium phosphate buffer (pH 5.8), 1.5 mM MgSO_4_, 2.0 mM Ca(NO_3_)_2_, 3.0 mM KNO_3_, 67 μM Na_2_EDTA, 8.6 μM FeSO_4_, 10.3 μM MnSO_4_, 30 μM H_3_BO_3_, 1.0 μM ZnSO_4_, 24 nM (NH_4_)_6_Mo_7_O_24_, 130 nM CoC1_2_, and 1 μM CuSO_4_) for 10 days. For S-deprived cultures, MgSO_4_ was replaced with an equimolar amount of MgCl_2_ to maintain the level of magnesium.

Tobacco (*Nicotianata bacum* cv. SamSun) plants were grown in a growth room at 25 °C under a 16 h light/8 h dark photoperiod. Drought and salt stress treatments were performed as follows. Seeds of the T_2_ generation of tobacco plants overexpressing *GmSULTR1;2b* and control plants were germinated on solid MS medium containing 50 mg L^−1^ hygromycin. After 10 days, the seedlings were transplanted onto solid MS medium containing 200 mM mannitol for osmotic treatment (drought stress) or 200 mM NaCl for salt treatment (salt stress) and maintained for 4 weeks. Ten biological replicates were used, and each one was measured for three repetitions. The sulfur deficiency treatment was performed as follows. T_2−_generation tobacco plants overexpressing *GmSULTR1;2b* and control plants were grown under normal conditions for 2 weeks and then cultured hydroponically in MGRL medium with 1.5 mM MgSO_4_ (+S) or 0 mM MgSO_4_ (−S) for 2 weeks. The tobacco plants were then transferred for 1 month to vermiculite irrigating MGRL medium containing 1.5 mM MgSO_4_ or 0 mM MgSO_4_. Three biological replicates were performed, and each one was measured for three repetitions.

### Expression analysis of *GmSULTR* genes

Total RNA was extracted from different tissues of soybean and tobacco plants using a Total RNA Plant Extraction Kit (Tiangen, Beijing, China). First-strand cDNAs were synthesized using the SuperScript III First-Strand Synthesis System (Invitrogen, Carlsbad, CA, USA). The semi-quantitative RT-PCR method was applied for expression analysis of *GmSULTRs* in soybean different tissues. All primers are provided in Additional file 5: Table S7. *GmTubulin* (GenBank: AY907703) was used as the endogenous reference gene for RT-PCR.

Real-time PCR of *GmSULTR1;2b* in soybean was performed using SYBR RT-PCR Mix (Invitrogen, USA) with an ABI 7500 system (Applied Biosystems, Foster City, CA, USA) and sequence-specific primers for *GmSULTR1;2b* combined with a 3′-fluorogenic minor groove binder (MGB) probe (Additional file 5: Table S7 and Figure S6). *GmTubulin* (GenBank: AY907703) expression was used as an internal control. For analysis of *GmSULTR1;2b* expression in soybean seedlings suffering from sulfur deficiency, normalized expression levels of *GmSULTR1;2b* were calculated using the formula ΔΔCT = (CT, _Target_ − CT, _Tubulin_) _x mM MgSO4_ − (CT, _Target_ − CT, _Tubulin_) _1.5 mM MgSO4_ [60]. Three biological and technical replicates were performed, and the data were analyzed using the ABI 7500 system v1.4.0 (Applied Biosystems).

We used RNA-seq data (GSE29163) for 10 soybean tissues (whole seeds at the globular (G.S), heart (H.S), cotyledon (C.S), early-maturation (E.S) and dry (D.S) stages of seed development and leaves (L), roots (R), stems (S), seedlings (SDL) and floral bud (F) tissues) downloaded from the GEO database for *in silico* expression analysis. The RPKM method was employed to estimate the expression levels of the 28 SULTR genes. The data were adjusted by the median-center genes and clustered by the centroid linkage hierarchical method using the Pearson correlation in Gensis1.7.6 [61].

### Full-length sequence cloning of *GmSULTR1;2b*

Total genomic DNA was extracted from the bulked leaves of 6–8 soybean seedlings using Plant Genomic DNA Kit (Tiangen, Beijing) following the manufacturer’s instructions. Specific *GmSULTR1;2b* primers were designed based on the genomic sequence, and the sequences are listed in Additional file 5: Table S7. Full-length *GmSULTR1;2b* was amplified from leaf cDNA and genomic DNA using specific primers.

### Yeast complementation analysis of the *GmSULTR1;2b* gene

The coding sequence of the *GmSULTR1;2b* cDNA was amplified by PCR using specific primers Y-GmSULTR1;2b-*Eco*R I F and Y-GmSULTR1;2b-*Not* I R containing a 5′ *Eco*R I and 3′ *Not* I oligonucleotide linker (Additional file 5: Table S7). The amplified fragment was inserted into the *Eco*R I -*Not* I site of the yeast expression vector p112A1NE. The p112A1NE plasmid containing *GmSULTR1;2b* (Yp112-*GmSULTR1;2b*) or *AtSULTR1;2* (Yp112-*AtSULTR1;2*) or the empty vector was transformed into CP154-7A (*Mat*, *his3*, *leu2*, *ura3*, *ade2*, *trp1*, *sul1::LEU2*, and *sul2::URA3*) [33] using the lithium acetate method [62]. Transformants were selected for tryptophan prototrophy on yeast nitrogen base (YNB) medium containing 20 g L^−1^ glucose and essential amino acids. Complementation of CP154-7A was examined for 3 days at 30 °C on YNB medium containing 0.1 mM sodium sulfate as the sole sulfur source. To measure the yeast growth rate, cells were incubated in YNB medium containing 0.1 mM sodium sulfate for 24 h at 30 °C. To determine the dependence of sulfate uptake on pH, the pH of YNB medium containing 0.1 mM sodium sulfate was adjusted by the addition of phosphate-buffered saline (PBS) to values ranging from 4.0 to 8.0, and the cells were grown at 30 °C for 18 h. Uptake of ^35^S-labeled sulfate by yeast cells was measured according to a previously described method [32] using a Beckman LS 6500 scintillation counter. Three replicates were performed. The *K*_m_ value was calculated by fitting the Michaelis-Menten equation, $\mathrm{y}$= *V*_max_ × $x$/(*K*_m_ + $x$), using Sigmaplot version 10.0 (Systat Software, Inc.).

### Transformation of *GmSULTR1;2b* into tobacco plants

An expression vector was constructed using Gateway Technology with Clonase™ II Kit (Invitrogen, USA). The open reading frame (ORF) of *GmSULTR1;2b* was obtained by PCR using the specific primers attB1-clone-1;2b F and attB1-clone-1;2b R (Additional file 5: Table S7). The amplified ORF containing the attB fragment was inserted into the expression vector pMDC83, which harbors a double CaMV 35S promoter and hygromycin resistance gene, resulting in 35S:*GmSULTR1;2b*. pMDC83 was digested with the restriction endonucleases *Asc*I and *Spe*I and then ligated using T4 DNA ligase, which resulted in an empty vector. The plasmid containing *GmSULTR1;2b* or empty vector (as a control) was introduced into *Agrobacterium tumefaciens* strain EHA105 using the freeze-thaw method, and *N. tabacum* was transformed by the leaf disk method. Transgenic tobacco plants containing *GmSULTR1;2b* or the empty vector were selected on 50 mg L^−1^ hygromycin and confirmed by PCR, RT-PCR and Southern blotting.

### Subcellular localization of the GmSULTR1;2b-GFP fusion protein

The GmSULTR1;2b ORF without the stop codon was inserted into the pMDC83-GFP vector, resulting in translational GFP fusion at the C-terminus of GmSULTR1;2b. This construct was transferred into onion epidermal cells by Gene-gun Bombardment. Cells harboring the empty pMDC83-GFP vector (35S:GFP) were used as a control. The GFP signals were monitored under a confocal spectral microscope (Leica CP SP2, Germany).

### Transformation of the *GmSULTR1;2b* promoter into soybean hairy roots

The promoter sequence of *GmSULTR1;2b* (approximately 2259 bp) was introduced into the pCAMBIA1381Z vector along with a β-glucuronidase (GUS) reporter gene, and the construct was transformed into soybean cotyledons by the freeze-thaw method using *Agrobacterium rhizogenes* cucumopine strain K599 to induce the growth of hairy roots. Soybean seeds were sterilized with chlorine gas for 6–8 h and then placed onto 1/2 MS solid medium for 5–6 days with a 16/8 h light/dark cycle at 25 °C for germination. Tissue inoculation was performed using the method of Savka [63]. Cotyledons were wounded on the abaxial side using a scalpel blade and then immersed in a culture of *A. rhizogenes* K599 harboring the recombinant vector. The processed tissues were cultured at 25 °C in the dark on MS medium containing carbenicillin (250 μg mL^−1^) and cefotetan (250 μg mL^−1^) with their abaxial sides facing upward. After 15–18 days, the hairy roots were used to assess *GUS* gene expression via histochemical staining according to a previously described method [64], with some modifications. The plant materials were washed with ultra-pure water at room temperature and immersed in X-Gluc solution (100 mM sodium phosphate buffer (pH 7.0), 10 mM EDTA, 0.1 % (*v/v*) Triton X-100, 1 mM K_3_[Fe(CN)_6_], 1 mM K_4_[Fe(CN)_6_] and 0.8 mg mL^−1^ 5-bromo-4-chloro-3-indolyl glucuronide) for 12 h at 37 °C in the dark. The staining solution was then removed, and the tissues were dehydrated in an ethanol series of 70, 90, and 100 % for 30 min each. GUS staining was observed, and images were obtained under a stereomicroscope (Olympus SZX12, Japan).

### Determination of sulfur-containing compounds and measurement of tobacco plant biomass

Total thiols were extracted by homogenizing tobacco leaves in 1:9 (*w/v*) ice-cold 0.9 % NaCl. The supernatant was assayed according to an improved previously described method [65]. The supernatant (1 mL) was added to 1 mL of methyl aldehyde (*φ* = 0.03) at pH 8.0. After standing for 5 min, 0.25 mL of 5, 5′-dithio bis-(2-nitrobenzoic acid) (DTNB) was added to the sample, which was placed in a water bath at 25 °C for 5 min. The absorbance at 412 nm was then measured. The Cys content of functional leaves from 2-month-old control and *GmSULTR1;2b*-overexpressing tobacco plants was measured by reverse-phase HPLC following a previously described method [66]. The concentration of amino acids was determined using o-phthalaldehyde, followed by fluorescence measurement at 335/447 nm. The composition of amino acids was determined by separating a 66 nmol sample of total free amino acids using an Agilent 1200 HPLC system (ZORBAX SB, RRHT C18, 150 mm × 4.6 mm, 3.5 lm columns). The sulfur content of plants was analyzed using an ICP-AES analyzer (Varian, VISTA-MPX, USA) following a previously described method [67]. T_2_*GmSULTR1;2b*-overexpressing tobacco plants and control plants were dried in a hot-air oven at 105 °C for 0.5 h and then at 80 °C for 72 h, and their dry weights were determined using a digital balance. Sulfate contents were determined according to the turbidimetric method [68].

The seeds from T_2_*GmSULTR1;2b*-overexpressing tobacco plants and control plants were harvested at maturity and dried at 28 °C; 1000 seeds from each plant were counted and weighed using a digital balance. The soluble protein content of the samples was measured according to the Bradford method [69], and the chlorophyll content was measured according to a previously described method [70]. Three biological replicates were performed and average values were calculated from three plants of each line. Statistical analysis was performed using SigmaPlot 10.0 (Systat Software, Inc.). Student’s *t*-test was used to assess the significance of differences between control tobacco plants and those overexpressing *GmSULTR1;2b*.

### Microarray analysis of gene expression

RNA was isolated from whole leaves of control and *GmSULTR1;2b* (S26 line)-overexpressing tobacco plants grown at 25 °C under normal conditions with a 16 h light/8 h dark photoperiod for 8 weeks and used for microarray analysis. The Agilent Tobacco Gene Expression Microarray Chip analysis was performed by Capital Bio Corporation (Beijing, China). Arrays were scanned with a confocal LuxScan™ scanner, and the images obtained were analyzed using the LuxScan 3.0 software. For the extraction of data from individual channels, spots with intensities of less than 400 units after subtraction of the background in both channels (Cy3 and Cy5) were removed. Space-dependent and intensity-dependent normalization based on a locally weighted scatterplot smoothing method (LOWESS) was employed. Transcripts with changes in expression of at least 2-fold were defined as differentially expressed. Gene annotations were acquired from NCBI (http://www.ncbi.nlm.nih.gov/) and TIGR (http://plantta.jcvi.org/search.shtml). Gene ontology was analyzed by MAS 3 (CapitalBio), and functional hierarchy analysis was performed by KEGG (http://www.genome.jp/kegg).

## Additional files

Additional file 1: Table S1. Information concerning the 28 soybean SULTR genes. **Table S2.** Accession numbers of putative SULTR protein sequences. **Figure S1.** *In silico* expression profiling of 28 soybean SULTR genes in 10 soybean tissues. **Figure S2.** Alignment of SULTR protein sequences. **Figure S3.** Subcellular localization of GmSULTR1;2b. **Figure S4.** RT-PCR assay of *GmSULTR1;2b* in different tissues of GmSULTR1;2b-overexpressing tobacco plants. **Figure S5.** Chlorophyll content of the leaves of *GmSULTR1;2b*-overexpressing and control tobacco plants grown under +S conditions. (DOCX 909 kb) Additional file 2: Table S3. Annotations of 227 genes with changes in expression levels greater than 2-fold. (XLSX 30 kb) Additional file 3: Table S4. Confirmation of microarray data by qPCR. **Table S5** Primer pairs used in confirming the reliability of the microarray results. (DOCX 14 kb) Additional file 4: Table S6. The 123 KEGG pathways associated with the 219 genes. (XLSX 17 kb) Additional file 5: Table S7. Primers used in this study. **Figure S6.** Standard curves generated for the amplification of *GmSULTR1;2b* and *Gmtubulin*. (DOCX 558 kb)

## Abbreviations

C: carbon
Cys: cysteine
DTNB: 5, 5′-dithio bis-(2-nitrobenzoic acid)
GSH: glutathione
GUS: β-glucuronidase
HPLC: high-performance liquid chromatography
ICP-AES: inductively coupled plasma-atomic emission spectrometry
KEGG: Kyoto encyclopedia of genes and genomes
*K*_m_: Michaelis-Menten constant
Met: methionine
MSD: membrane-spanning domain
N: nitrogen
NCBI: National Center for Biotechnology Information
ORF: open reading frame
PBS: phosphate-buffered saline
S: sulfur
SAHH: S-adenosylhomocysteine hydrolase
STAS: sulfate transporter anti sigma factor antagonist domain
SUL: yeast sulfate transporter
SULTR: plant sulfate transporter
TIGR: The Institute for Genomic Research
YNB: yeast nitrogen base
