# Supplementary material for: Identification and functional characterization of the sulfate transporter gene GmSULTR1;2b in soybean
Source: BMC Genomics. 2016 May 20;17:373. doi: 10.1186/s12864-016-2705-3 (PMC4874011; doi:10.1186/s12864-016-2705-3)
Supplement: Additional file 1: Table S1. — Information concerning the 28 soybean SULTR genes. Table S2. Accession numbers of putative SULTR protein sequences. Figure S1. In silico expression profiling of 28 soybean SULTR genes in 10 soybean tissues. Figure S2. Alignment of SULTR protein sequences. Figure S3. Subcellular localization of GmSULTR1;2b. Figure S4. RT-PCR assay of GmSULTR1;2b in different tissues of GmSULTR1;2b-overexpressing tobacco plants. Figure S5. Chlorophyll content of the leaves of GmSULTR1;2b-overexpressing and control tobacco plants grown under +S conditions. (DOCX 909 kb) [file 12864_2016_2705_MOESM1_ESM.docx]

**Table S1** The information concerning the 28 soybean SULTR genes

| Gene name | Locus ID | Gene length (bp) | cDNA length (bp) | No. of introns | Protein length (AA) | pI | Mol. wt.(kDa) | ChrNo. |
| --- | --- | --- | --- | --- | --- | --- | --- | --- |
| *GmSULTR1;1a* | Glyma04g11580 | 6894 | 1833 | 13 | 634 | 9.92 | 69.47 | 4 |
| *GmSULTR1;1b* | Glyma06g11140 | 4232 | 2316 | 12 | 661 | 9.38 | 72.56 | 6 |
| *GmSULTR1;2a* | Glyma08g19240 | 5187 | 2444 | 12 | 657 | 9.84 | 71.96 | 8 |
| *GmSULTR1; 2b* | Glyma15g05760 | 4896 | 2514 | 12 | 659 | 9.84 | 72.19 | 15 |
| *GmSULTR1;3a* | Glyma13g02060 | 5684 | 2605 | 12 | 658 | 9.54 | 72.23 | 13 |
| *GmSULTR1;3b* | Glyma14g34220 | 5885 | 2347 | 10 | 605 | 9.55 | 66.10 | 14 |
| *GmSULTR2;1a* | Glyma08g14700 | 7103 | 2294 | 11 | 664 | 10.04 | 72.27 | 8 |
| *GmSULTR2;1b* | Glyma11g36210 | 8042 | 2603 | 11 | 653 | 9.87 | 70.96 | 11 |
| *GmSULTR2;1c* | Glyma18g02240 | 6881 | 2444 | 11 | 653 | 9.86 | 70.99 | 18 |
| *GmSULTR2;2a* | Glyma06g11150 | 4846 | 1962 | 11 | 653 | 9.8 | 71.25 | 6 |
| *GmSULTR2;2b* | Glyma14g34210 | 5899 | 2844 | 11 | 654 | 10.05 | 71.49 | 14 |
| *GmSULTR2;2c* | Glyma13g02080 | 12351 | 2821 | 11 | 654 | 9.93 | 73.40 | 13 |
| *GmSULTR2;3* | Glyma18g02230 | 7356 | 2452 | 11 | 668 | 9.87 | 72.27 | 18 |
| *GmSULTR3;1a* | Glyma03g31310 | 8924 | 2521 | 11 | 656 | 9.46 | 72.66 | 3 |
| *GmSULTR3;1b* | Glyma19g34160 | 9180 | 2760 | 11 | 656 | 9.47 | 72.61 | 19 |
| *GmSULTR3;2a* | Glyma02g16365 | 8255 | 2579 | 9 | 657 | 9.42 | 72.93 | 2 |
| *GmSULTR3;2b* | Glyma10g03460 | 10497 | 2468 | 11 | 657 | 9.33 | 72.92 | 10 |
| *GmSULTR3;3a* | Glyma07g34370 | 7206 | 2777 | 12 | 644 | 9.85 | 70.04 | 7 |
| *GmSULTR3;3b* | Glyma20g02080 | 7492 | 2624 | 12 | 658 | 9.69 | 71.73 | 20 |
| *GmSULTR3;3c* | Glyma07g34360 | 8244 | 2728 | 12 | 656 | 9.21 | 71.65 | 7 |
| *GmSULTR3;4a* | Glyma07g00840 | 4188 | 2358 | 12 | 648 | 9.86 | 69.91 | 7 |
| *GmSULTR3;4b* | Glyma08g22120 | 4336 | 2385 | 12 | 663 | 9.93 | 73.10 | 8 |
| *GmSULTR3;4c* | Glyma13g43670 | 5407 | 2466 | 12 | 649 | 9.95 | 71.39 | 13 |
| *GmSULTR3;4d* | Glyma15g01710 | 5550 | 2490 | 12 | 652 | 9.82 | 71.83 | 17 |
| *GmSULTR3;5a* | Glyma07g09710 | 5287 | 1999 | 11 | 621 | 6.77 | 68.88 | 7 |
| *GmSULTR3;5b* | Glyma09g32110 | 6110 | 2145 | 10 | 640 | 9.65 | 70.41 | 9 |
| *GmSULTR4;2* | Glyma02g10590 | 8263 | 2286 | 16 | 702 | 9.26 | 76.98 | 2 |
| *GmSULTR4;1* | Glyma18g52270 | 8159 | 2566 | 16 | 698 | 9.39 | 76.69 | 18 |

pI: isoelectric point; Mol wt: molecular weight; ChrNo: chromosome nomber

**Table S2** Accession numbers of SULTR protein sequences

| Species | Protein name | Protein Accession (GenBank) |
| --- | --- | --- |
| *Glycine max* | GmSultr1;1a | XP_003526594 |
| *Glycine max* | GmSultr1;1b | XP_003526594 |
| *Glycine max* | GmSultr1;2a | XP_003532966 |
| *Glycine max* | GmSultr1;2b | XP_003547605 |
| *Glycine max* | GmSultr1;3a | XP_003543770 |
| *Glycine max* | GmSultr1;3b | XP_006596866 |
| *Glycine max* | GmSultr2;1a | XP_003531364 |
| *Glycine max* | Gmsultr2;1b | XP_003538517 |
| *Glycine max* | Gmsultr2;1c | XP_003552820 |
| *Glycine max* | Gmsultr2;2a | XP_003526596 |
| *Glycine max* | Gmsultr2;2b | XP_003544185 |
| *Glycine max* | Gmsultr2;2c | XP_003543772 |
| *Glycine max* | Gmsultr2;3 | XP_006601860 |
| *Glycine max* | Gmsultr3;1a | XP_003521258 |
| *Glycine max* | Gmsultr3;1b | XP_003554265 |
| *Glycine max* | Gmsultr3;2a | XP_003518908 |
| *Glycine max* | Gmsultr3;2b | XP_003536673 |
| *Glycine max* | Gmsultr3;3a | XP_003529415 |
| *Glycine max* | Gmsultr3;3b | XP_003556073 |
| *Glycine max* | Gmsultr3;3c | XP_003528541 |
| *Glycine max* | Gmsultr3;4a | XP_003529722 |
| *Glycine max* | Gmsultr3;4b | XP_003531685 |
| *Glycine max* | Gmsultr3;4c | XP_003543650 |
| *Glycine max* | Gmsultr3;4d | XP_003546346 |
| *Glycine max* | Gmsultr3;5a | XR_136691 |
| *Glycine max* | Gmsultr3;5b | XR_416059 |
| *Glycine max* | Gmsultr4;1 | XP_003520027 |
| *Glycine max* | Gmsultr4;2 | XP_003552670 |
| *Arabidopsis* *thaliana* | AtSULTR1;1 | AB018695 |
| *Arabidopsis* *thaliana* | AtSULTR1;2 | AB042322 |
| *Arabidopsis* *thaliana* | AtSULTR1;3 | AB049624 |
| *Arabidopsis* *thaliana* | AtSULTR2;1 | AB003591 |
| *Arabidopsis* *thaliana* | AtSULTR2;2 | D85416 |
| *Arabidopsis* *thaliana* | AtSULTR3;1 | D89631 |
| *Arabidopsis* *thaliana* | AtSULTR3;2 | AB004060 |
| *Arabidopsis* *thaliana* | AtSULTR3;3 | AB023423 |
| *Arabidopsis* *thaliana* | AtSULTR3;4 | AB054645 |
| *Arabidopsis* *thaliana* | AtSULTR3;5 | AB061739 |
| *Arabidopsis* *thaliana* | AtSULTR4;1 | AB008782 |
| *Arabidopsis* *thaliana* | AtSULTR4;2 | AB052775 |
| *Oryza sativa* | OsSULTR1;1 | AF493790 |
| *Oryza sativa* | OsSULTR1;2 | XP_470587 |
| *Oryza sativa* | OsSULTR1;3 | AF493790 |
| *Oryza sativa* | OsSULTR2;1 | AAN59769 |
| *Oryza sativa* | OsSULTR2;2 | AAN59770 |
| *Oryza sativa* | OsSULTR3;1 | NP_921514 |
| *Oryza sativa* | OsSULTR3;2 | AAN06871 |
| *Oryza sativa* | OsSULTR3;3 | AK104831 |
| *Oryza sativa* | OsSULTR3;4 | BAD68396 |
| *Oryza sativa* | OsSULTR3;5 | NM_192602 |
| *Oryza sativa* | OsSULTR3;6 | NM_191791 |
| *Oryza sativa* | OsSULTR4;1 | AF493793 |
| *Populus tremula* × *P. alba* | PtaSULTR1;1 | DQ906929 |
| *Populus tremula* × *P. alba* | PtaSULTR1;2 | DQ174472 |
| *Populus tremula* × *P. alba* | PtaSULTR2;1a | DQ906931 |
| *Populus tremula* × *P. alba* | PtaSULTR2;1b | DQ906933 |
| *Populus tremula* × *P. alba* | PtaSULTR2;2 | DQ174473 |
| *Populus tremula* × *P. alba* | PtaSULTR3;1a | DQ174470 |
| *Populus tremula* × *P. alba* | PtaSULTR3;1b | DQ906928 |
| *Populus tremula* × *P. alba* | PtaSULTR3;2a | DQ174469 |
| *Populus tremula* × *P. alba* | PtaSULTR3;2b | DQ906934 |
| *Populus tremula* × *P. alba* | PtaSULTR3;3a | DQ906924 |
| *Populus tremula* × *P. alba* | PtaSULTR3;3b | DQ906926 |
| *Populus tremula* × *P. alba* | PtaSULTR3;4a | DQ174467 |
| *Populus tremula* × *P. alba* | PtaSULTR3;4b | DQ174466 |
| *Populus tremula* × *P. alba* | PtaSULTR3;5 | DQ906927 |
| *Populus tremula* × *P. alba* | PtaSULTR4;1 | DQ906930 |
| *Populus tremula* × *P. alba* | PtaSULTR4;2 | DQ906935 |


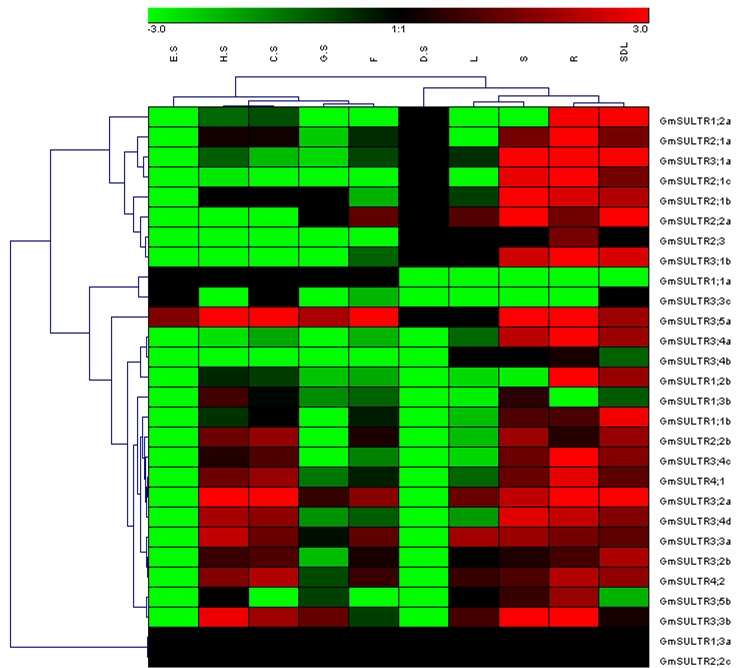


**Figure S1** Expression profiling *in silico* of 28 soybean SULTR genes for 10 soybean tissues

E.S, early-maturation stage whole seeds; H.S, heart stage whole seeds; C.S, cotyledon stage whole seeds; G.S, globular stage whole seeds; D.S, dry stage whole seeds; F, floral bud; L, leaves; R, root; S, stems; SDL, seedlings. The RPKM method was employed to estimate the expression levels of 28 SULTR genes. The data were adjusted by the median-center genes and clustered by the centroid linkage hierarchical method using the Pearson correlation in Gensis1.7.6.


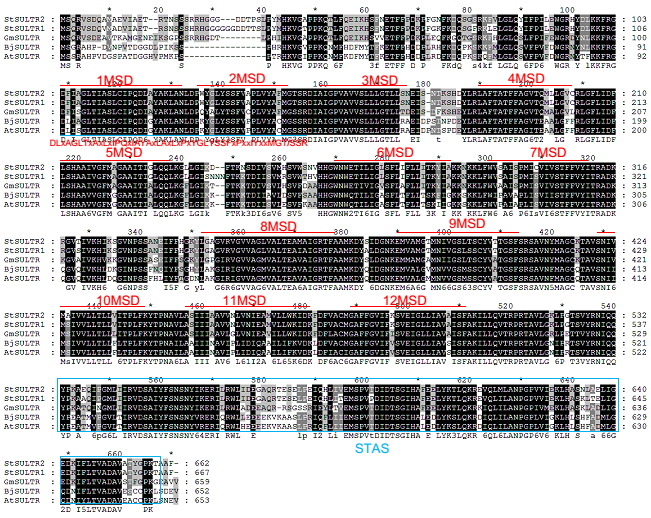
**Figure S2** Alignment of SULTR protein sequences

StSULTR1 (CAA57710) and StSULTR2 (CAA57711) are from *Stylosanthes hamata*. GmSULTR (GmSULTR1;2b) is from *Glycine max*. BjSULTR (BjSULTR1;2b AFX60924) is from *Brassica juncea*. AtSULTR (AtSULTR1;2, AEE36056) is from *Arabidopsis thaliana*. MSD, Membrane-spanning domain; STAS, Sulfate transporter anti sigma factor antagonist domain; blue underline represent DLxAGLTxAxLxIPQxIAYAxLAxLxPxYGLYSSFxPxxIYxxMGT/SSR motif.

**Figure S3** Subcellular localization of GmSULTR1;2b.

Photographs were taken with a confocal microscope.
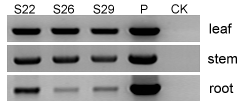


**Figure S4** RT-PCR assay of *GmSULTR1;2b* in different tissues of GmSULTR1;2b-overexpressing tobacco plants.

S22, S26 and S29, tobacco plants transformed with *GmSULTR1;2b*; P, plasmid containing *GmSULTR1;2b* as a positive control; CK, tobacco plant transformed with the pMDC83 empty vector as a negative control*.*


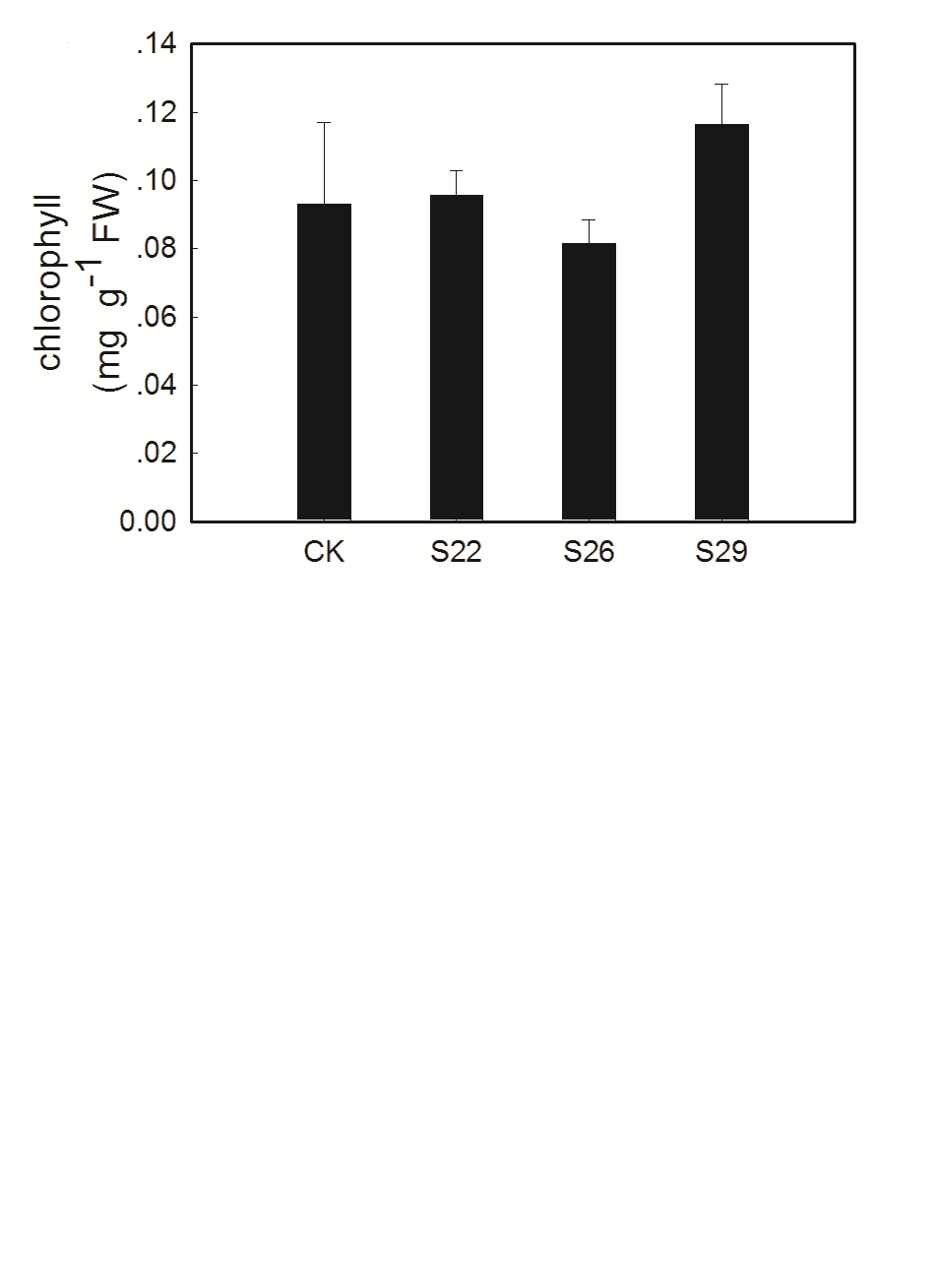


**Figure S5** Chlorophyll content of the leaves of *GmSULTR1;2b*-overexpressing tobacco plants and control plants grown under +S conditions for 2 weeks. Three biological replicates were performed. Error bars represent the standard deviation. CK, tobacco plant transformed with the pMDC83 empty vector as a control; S22, S26 and S29, tobacco plants transformed with *GmSULTR1;2b*.
