# Supplementary material for: Identification and functional characterization of the sulfate transporter gene GmSULTR1;2b in soybean
Source: BMC Genomics. 2016 May 20;17:373. doi: 10.1186/s12864-016-2705-3 (PMC4874011; doi:10.1186/s12864-016-2705-3)
Supplement: Additional file 3: Table S4. — Confirmation of microarray data by qPCR. Table S5 Primer pairs used in confirming the reliability of the microarray results. (DOCX 14 kb) [file 12864_2016_2705_MOESM3_ESM.docx]

**Table S4** Confirmation of microarray data by qPCR

| ProbeName | FC of microarray | FC of qPCR | Discription |
| --- | --- | --- | --- |
| A_95_P006336 | 0.13 | 0.12±0.03 | SAHH [ Populus trichocarpa(black cottonwood) ] |
| A_95_P042806 | 2.04 | 2.51±0.15 | 4-coumarate--CoA ligase-like 10 [ Arabidopsis thaliana ] |
| A_95_P110977 | 3.26 | 3.78±0.56 | OPT [Vitis vinifera] |
| A_95_P178392 | 0.46 | 0.49±0.02 | PSAH-1 [ Arabidopsis thaliana ] |
| A_95_P190077 | 0.45 | 0.42±0.01 | Divinyl ether synthase [Nicotiana tabacum] |
| A_95_P237579 | 9.06 | 9.44±0.25 | Putative pectate lyase Nt59 [Nicotiana tabacum] |
| A_95_P242602 | 3.48 | 2.53±0.17 | Nramp3 [ Arabidopsis thaliana ] |

FC, fold change

**Table S5** Primer pairs used in confirming the reliability of the microarray results

| ProbeName | PrimaryAccession | qPCR-forward | qPCR-reverse |
| --- | --- | --- | --- |
| A_95_P006336 | FG125386 | CTTTGCCTTTCTTTGGCTATT | TATCCAACCCGGTATCTTACG |
| A_95_P042806 | BP130453 | GAGGTCCAAATGTAACTAAACG | TGATATTTTCTCCCCTCCACG |
| A_95_P110977 | CV019992 | GTTTTCAAGTAGCCCACAAG | GCAAATCATTTACAGAGCAC |
| A_95_P178392 | DV999089 | GGCCATGACTCGAAATAATA | GCTGCCATACTAGACAAATC |
| A_95_P190077 | AF070976 | GCTCGACTTGCTAAAGAAAT | GGACCATGAAATCTTTTTTG |
| A_95_P237579 | FG195217 | CATTCTCTGCACCAATCTCT | GAAGGATCCACTGCTATCAC |
| A_95_P242602 | EB427401 | CAAAACCTAATGGAGTTGAA | ATCTCTCTGGACTGCACTAG |
