# Supplementary material for: Identification and functional characterization of the sulfate transporter gene GmSULTR1;2b in soybean
Source: BMC Genomics. 2016 May 20;17:373. doi: 10.1186/s12864-016-2705-3 (PMC4874011; doi:10.1186/s12864-016-2705-3)
Supplement: Additional file 5: Table S7. — Primers used in this study. Figure S6. Standard curves generated for the amplification of GmSULTR1;2b and Gmtubulin. (DOCX 558 kb) [file 12864_2016_2705_MOESM5_ESM.docx]

**Table S7** Primers used in this paper

|  | Primer name | Sequence (5’→3’) |
| --- | --- | --- |
| Specific primers were used for the full-length sequence cloning of *GmSULTR* 1;2b. | clone-GmSULTR1;2b F | ATGAGTCAGCGTGTGAGTGACGAGG |
|  | clone-GmSULTR1;2b R | TCATACTACTGCTTCACCCTTTGGACC |
| Semi-quantitative RT-PCR primers of *GmSULTRs* were used in soybean. | GmSULTR1;1b F | CCGTTACCGACATAGACAC |
|  | GmSULTR1;1b R | CTGTAGCAGAATCGGATAGG |
|  | GmSULTR1;2a F | GCATCCATGCTTTTGAAGA |
|  | GmSULTR1;2a R | TTTGTACATCAACTGGGAC |
|  | GmSULTR1;2b F | CACTAGTGGCATCCATGCT |
|  | GmSULTR1;2b R | CATCAACTGGGGCTATAAC |
|  | GmSULTR1;3a F | GATGGTCGAGATGTCACCCGTT |
|  | GmSULTR1;3a R | CTTGGGTTTAAATTCGAGTGAG |
|  | GmSULTR2;3 F | CAACACTAGGTTCCTAAAATGTG |
|  | GmSULTR2;3 R | TGAATAGGAACTTTCAATGTTTG |
|  | GmSULTR3;4d F | GGAAAGAGAAGTTAAAGGAAC |
|  | GmSULTR3;4d R | GGCATCAGCTGATCAACCTGAGA |
|  | GmSULTR4;2 F | TCATTAGAAGACAAACCAAG |
|  | GmSULTR4;2 R | CATTATTTGAAAGAATGGCT |
|  | GmTublin F | GGAGTTCACAGAGGCAGAG |
|  | GmTublin R | CACTTACGCATCACATAGCA |
| Quantitative RT-PCR primers of *GmSULTR1;2b* were used in soybean. | qPCR-GmSULTR1;2b F | AATATGCACTCTACACCAGTTTTGTGT |
|  | qPCR- GmSULTR 1;2b R | GGGTCCTATGGCAATATC |
|  | qPCR-1;2b TaqMan MGB PROBE | ATATTGCCATAGGACC |
| Primers were used in constructing yeast recombinant vector. | Y-GmSULTR1;2b-*Eco*RⅠF | GGAATTCATGAGTCAGCGTGTGAGTGACGAGG |
|  | Y- GmSULTR1;2b*-Not*ⅠR | GGCGGCCGCTCATACTACTGCTTCACCCTTTGGACC |
| Primers were used in constructing plant recombinant vector. | attB1-clone- ;2b F | GGGGACAAGTTTGTACAAAAAAGCAGGCTTCGACTCCTTTATGGATAATAATGAGTCAGCGTGTGAGTGACGAGG |
|  | attB2-clone-1;2b R | GGGGACCACTTTGTACAAGAAAGCTGGGTCCGATAACAAATTGCAGGCATAGTACTACTGCTTCACCCTTTGGACC |


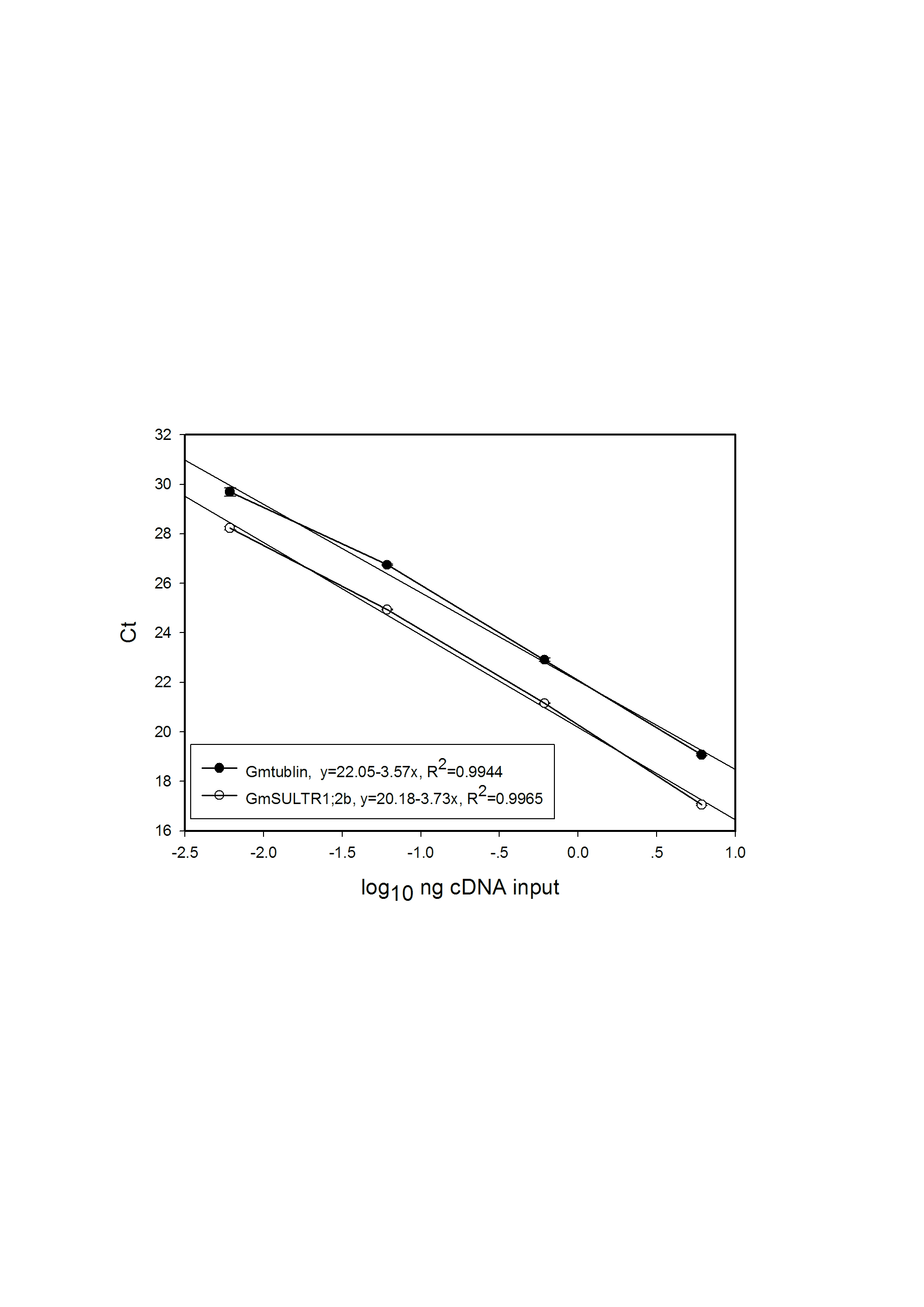


**Figure S6** Standard curves generated for the amplification of *GmSULTR1;2b* and *Gmtubulin*.

The plot of Ct vs the log_10_ of target cDNA input represents a straight line, which can be described by the corresponding regression formula.
